# Air-assisted cloud point extraction coupled with inductively coupled plasma optical emission spectroscopy for determination of samarium in environmental samples

**DOI:** 10.1007/s44211-022-00181-9

**Published:** 2022-08-31

**Authors:** Wael I. Mortada, Aya A. Awad, Mohamed M. El-Defrawy, Magdi E. Khalifa

**Affiliations:** 1 Urology and Nephrology Center, Mansoura University, Mansoura, 35516 Egypt; 2 Chemistry Department, Faculty of Science, Mansoura University, Mansoura, Egypt

**Keywords:** Air-assisted cloud point extraction, Samarium, Inductively coupled plasma-optical emission spectroscopy, Environmental samples

## Abstract

For the first time, air-assisted cloud point extraction (AACPE) was presented to preconcentrate metal ions. The procedure was conjugated with inductively coupled plasma-optical emission spectroscopy for determination of samarium. In this procedure, samarium ions were complexed with aluminon and extracted into Triton X-114 in the presence of potassium iodide. The mixture was repeatedly sucked and dispersed with a syringe (three times) to create cloud solution. Experimental factors that affect the extraction competence of the AACPE procedure, such as pH, amount of aluminon and Triton X-114, salt addition, number of suction/injection cycles, and centrifugation rate and time, have been investigated and optimized. A linear calibration curve from 0.2 to 200.0 μg L^−1^ with enrichment factor and detection limit of 102 and 0.06 μg L^−1^, respectively, was established under the optimum experimental conditions. The approach was used to determine samarium in wastewater and rock samples, with recoveries ranging from 98% to 99%.

**Graphical abstract:**

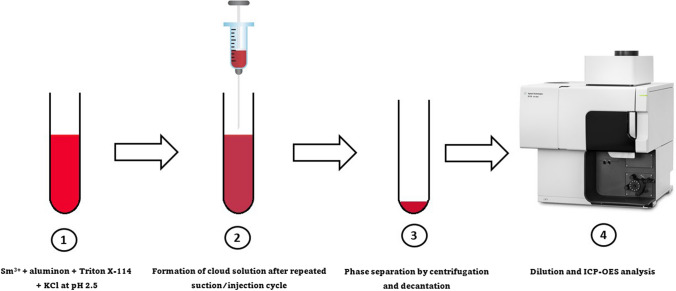

## Introduction

Samarium (Sm) exists naturally in many minerals such as monazite, bastnasite, and samarskite along with other rare earth elements. Samarium iodide (SmI_2_) and samarium oxide (Sm_2_O_3_) are used as catalysts. Samarium oxide is also used for the production of optical and infrared adsorbing glass. Samarium cobalt alloy (SmCo_5_) is used in making powerful magnets [1].

The most accurate techniques for determining Sm are neutron activation analysis (NAA) [2], inductively coupled plasma-mass spectrometry (ICP-MS) [3], and inductively coupled plasma-optical emission spectrometry (ICP-OES) [4]. Generally, the direct measurement is limited due to background interference from sample matrix. To overcome this problem, several extraction procedures are used including solid phase extraction (SPE) [5], liquid–liquid extraction [6] and precipitation [7].

In the past few decades, interest in environmentally friendly separation procedures has increased. Among them microextraction procedures have performed well to improve separation, achieve high efficiency, and reduce environmental hazard effects caused by the use of toxic organic solvents [8, 9]. Examples of eco-friendly extraction procedures are cloud point extraction (CPE) [10–13], dispersive liquid–liquid microextraction (DLLME) [14] and solidification of floated organic drop [15].

The characteristics of CPE include simplicity, high efficiency, safety, and versatility [16]. It is based on the analyte being extracted from the aqueous sample and introduced into a phase with a high surfactant content [17]. Because the use of organic extractants has been replaced by surfactants in CPE, it becomes eco-friendly technique [18]. The procedure was used for extracting of many *f*-block elements [19–22]. The procedure was applied to extract Sm^3+^ using various complexing agents including Alizarin Red S [19], diglycolamide [21], 8-hydroxyquinoline [23], and 1-(2-thenoyl)-3,3,3-trifluoracet [24].

Recently, a new approach known as air-assisted CPE (AACPE) has been developed for preconcentration of heterocyclic aromatic amines before analysis by high performance liquid chromatography [25]. The procedure uses air agitation, as a dispersion tool, in conjunction with conventional CPE to improve extraction efficiency and expedite the rapid extraction process. The strategy combines the benefits of both DLLME and the CPE [26].

Aluminon, ammonium salt of aurintricarboxylic acid, is a triphenyl methane derivative that have important applications. It is used as a colorimetric reagent for aluminum, acid–base indicator [27]. Medically, the compound and its derivatives can be used as antiviral [28] and inhibitor for apoptosis [29]. Despite the fact that it forms stable complexes with a wide range of metal ions, it has received little attention as a complexing agent in separation processes [30].

In this study, the AACPE was applied, for the first time, to preconcentrate metal ions prior to determination by ICP-OES. Samarium was selectively extracted into Triton X-114 at pH 2.5 after complexation with aluminon. The dispersion process is enhanced by air agitation using a syringe. Various experimental parameters that influenced extraction efficiency were studied. The approach was employed for quantification of Sm in wastewater and rock samples.

## Experimental

### Apparatus

A Genway 7300 spectrophotometer (Cole-Parmer Ltd., Staffordshire, UK) was used to record UV–visible spectra. ICP-OES analysis was performed with an Agilent 5100 ICP-OES (Agilent Technologies, Melbourne, Australia). Table 1 shows the operating conditions of ICP-OES for Sm^3+^ determination. A digital pH meter was used to take the readings (Hanna Instruments Inc, RI, USA). To speed up the phase separation, a commercial centrifuge (Hinotek Technology Co., Ningbo, China) was used.

**Table 1:** ICP-OES operating conditions for analysis of samarium

*Rf* generator power	Plasma gas flow rate	Auxiliary gas flow rate	Nebulizer gas flow rate	Delay time	Integration time	Wavelength
1200 W	12 L min^−1^	1.0 L min^−1^	0.7 L min^−1^	15 s	3 s	359.160 nm

### Chemicals

Ultrapure chemicals were used in the study and purchased from Merck (Darmstadt, Germany) or Sigma-Aldrich (St. Louis, MO, USA). The stock standard solution of Sm^3+^ (1000 mg L^−1^) was prepared by dissolving 0.2956 g of Sm(NO_3_)_3_·6H_2_O (99.9%) in 5.0 mL of HNO_3_ (1.0 mol L^−1^) and the volume was made up to 100 mL with double distilled water. The stock solution of aluminon (10^–2^ mol L^−1^) was prepared by dissolving 0.4734 g in 100 mL of double distilled water. The pH was controlled using the following solutions: HCl/KCl (pH 1.0–2.0), acetate buffer (pH 3.0–6.0), and hexamine buffer (pH 7.0–8.0). Hard Rock Mine Waste standard reference material (SRM 2780a) from the National Institute of Standards and Technology (NIST, Gaithersburg, MD, USA) was processed to evaluate the accuracy.

### General procedure for AACPE

To aqueous solution of standard or sample, 2.0 mL buffer solution (pH 2.5), 0.5 mL of 10^–2^ mol L^−1^ aluminon, 1.0 mL of 5.0% (v/v) Triton X-114 and 1.0 mL of 1 mol L^−1^ KCl were added in the same sequence and the volume was adjusted to 50 mL by double distilled water. The contents were then rapidly sucked into a 50-mL syringe and injected into the tube (3 times) using a needle. To increase the viscosity of the surfactant-rich phase, the tubes were first placed in ice bath for 10 min before being centrifuged at 3000 rpm for 5 min. The upper aqueous phase was decanted, and the residual surfactant-rich phase was removed by micro-syringe and its volume was made up to 0.5 mL by 1.0 mol L^−1^ of HNO_3_ prior to aspiration into ICP-OES. The extraction recovery (*R*), which may be computed using the following formula, was used to measure the extraction efficiency.

1 $$ R(\% ) = \frac{C_s V_s }{{C_i V_i }} \times 100 $$

where *C*_*s*_ is the concentration of Sm^3+^ in the surfactant-rich phase of volume *V*_*s*_, *C*_*i*_ is its initial concentration and *V*_*i*_ is the initial volume.

### Environmental samples

Rock samples were taken from Abu-Tartour phosphate mine (New Valley, Egypt) and collected in polyethylene bags. Rock samples were crushed and powdered to less than 120 mesh using agate mortar. In Teflon vessels, accurately weighed samples or SRM (0.2–0.3 g) were mixed with a mixture of HF, HNO_3_ and double distilled water (2.0 mL each). The microwave digestion was proceeded based on our previously optimized program: 145 °C (5 min), 165 °C (5 min) and 170 °C (20 min) [31]. Following cooling, 20 mL of boric acid (5% w/v) was added to neutralize the excess HF and the volume was completed to 50 mL with double distilled water. Industrial wastewater samples, from Sinmar Chemicals Factory (Port-Said, Egypt), were collected in acid-washed polyethylene vessels. The water samples were filtered through 0.45 μm cellulose nitrate membrane (Millipore, Bedford, MA, USA), acidified to pH 2 with HNO_3_.

## Results and discussion

### Stoichiometry of the complex

**Fig. 1:**
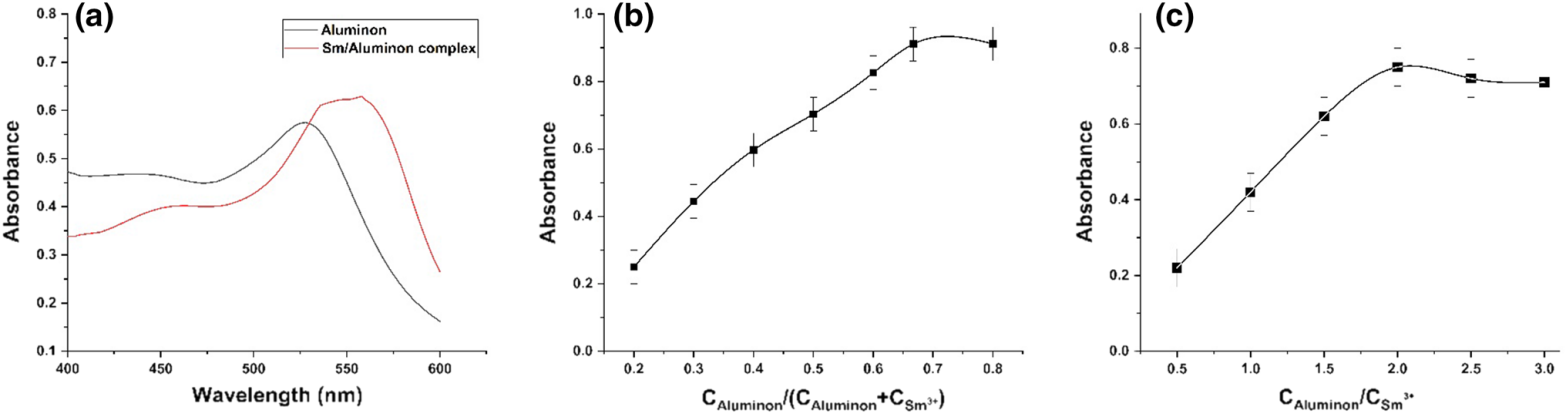
**a** UV–Vis spectra of aluminon and its samarium complex, **b** Job’s method of continuous variation, **c** mole ratio plot

The UV–Vis spectrum of samarium-aluminon complex shows maximum absorbance at 557 nm (Fig. 1a). The stoichiometry of the complex was evaluated using continuous variation and mole-ratio methods. As indicated in Fig. 1b, maximum absorbance was achieved at the ratio between *C*_Aluminon_ and *C*_Aluminon_ + *C*_Sm_ equal 0.67 suggesting a 1:2 metal ligand complex. This finding was confirmed by an inflection at *C*_Aluminon_/*C*_Sm_ of 2.0 in molar ratio plot (Fig. 1c).

### Optimization of AACPE procedure

#### Effect of pH

**Fig. 2:**
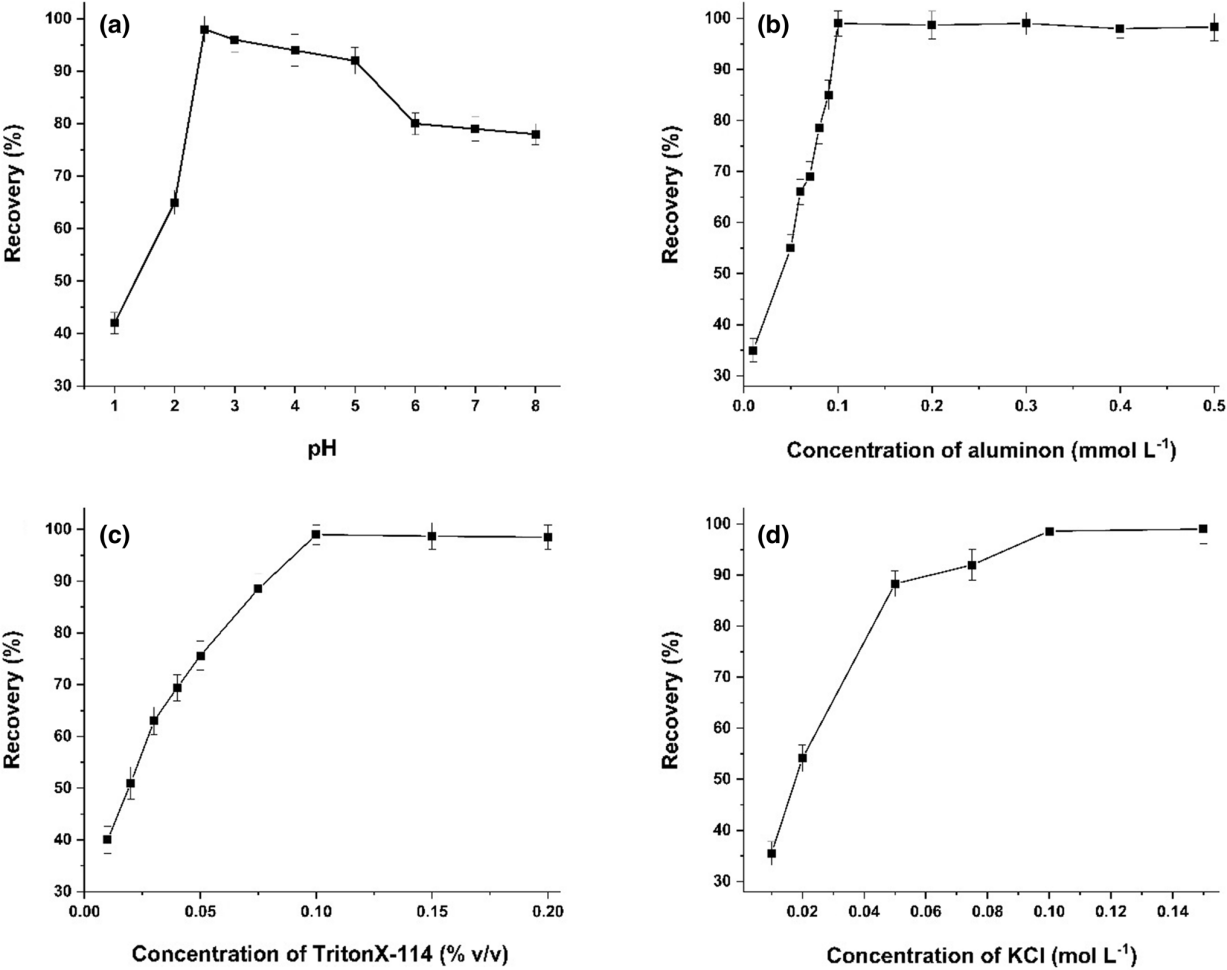
Effect of **a** pH, **b** concentration of aluminon, **c** amount of Triton X-114, **d** concentration of KCl on the AACPE of Sm^3+^

The effect of sample pH on AACPE of Sm^3+^ was investigated within the range of 1.0–8.0. The results in Fig. 2a showed that Sm^3+^ was separated at quantitative value (˃95%) in the pH range 2.5–5.0. The extraction was slightly decreased at higher pH because of the possible hydrolysis of the metal ions. As a result, the optimum pH was selected as 2.5 during the experiments.

#### The effect of aluminon concentration

The influence of aluminon concentration on Sm^3+^ extraction by the suggested procedure was studied from 0.01 to 0.5 mmol L^−1^. The results shown in Fig. 2b illustrated that the maximum extraction was attained when the concentration of aluminon was 0.1 mmol L^−1^ or more. Therefore, 0.1 mmol L^−1^ aluminon was defined as the optimum concentration for the AACPE of Sm^3+^ during this work.

#### The effect of Triton X-114 concentration

Triton X-114 is a nonionic surfactant that is widely employed in CPE due to its benefits, which include commercial availability in pure form, low cost and toxicity, high density, which makes phase separation by centrifugation easier, and a relatively low cloud point temperature [32]. Figure 2c displayed the impact of Triton X-114 concentration on extraction efficiency of the presented procedure. As shown, increasing the amount of TritonX-114 to from 0.01 to 0.1% (v/v) increased the extraction of Sm^3+^ and the further increase did not improve the extraction. As a result, for subsequent experiments, 0.1% (v/v) of Triton X-114 was used.

#### Salting-out effect

Strong electrolytes facilitate dehydration of Triton X-114’s poly(oxyethylene) chains, which aids phase separation by raising the density of the aqueous phase and reducing the surfactant's cloud point temperature [33, 34]. The effect of salt concentration on AACPE of Sm^3+^ was evaluated using KCl as a salting-out agent. The obtained data denoted that the extraction was enhanced at room temperature by addition of KCl and accomplished a plateau at concentration of 0.1 mol L^−1^.

#### Effect of number of suction/injection cycles

**Fig. 3:**
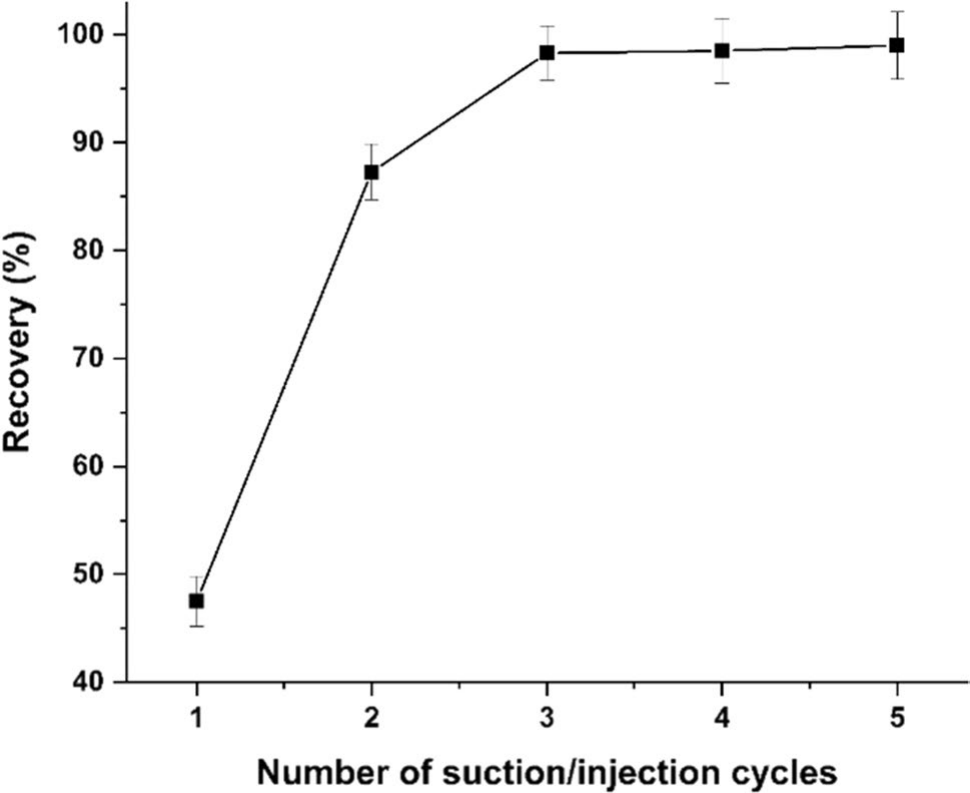
Effect of number of suction/injection cycles of the extraction of Sm^3+^

In the present study, the mixture of analyte, complexing agent, Triton X-114 and KCl was rapidly withdrawn into a 50 mL syringe and then injected into the tube. It is observed that increasing the number of suction/injection cycles enhances the turbidity of the solution resulting from the dispersion of micelles in the aqueous phase. As a result, the effect of number of suction/injection cycles on the extraction of Sm^3+^ was investigated in the range of 1–5 cycles to achieve the optimum status. The results in Fig. 3 showed that increasing the number of suction/injection cycles increased the extraction efficiency until reached a plateau at the third cycle. Therefore, 3 suction/injection cycles were chosen for further experiments. This step was completed in less than 15 s, indicating that the extraction procedure is very rapid.

#### Centrifugation conditions

To attain quantitative extraction in a short period, the influence of centrifugation time was studied in the range from 3 to 10 min at 2000 to 3000 rpm. Table 2 indicated that perfect extraction was obtained by centrifugation at 3000 rpm for 5 or 10 min. Therefore, centrifugation for 5 min at 3000 rpm was adopted as optimum centrifugation conditions in the study.

**Table 2:** Effect of centrifugation rate and time on the AACPE of Sm^3+^

Centrifugation rate	Centrifugation time	Recovery of Sm^3+^ (%)
2000	3	65.4 ± 4.3
	5	73.2 ± 3.9
	10	82.0 ± 4.5
2500	3	76.2 ± 3.9
	5	83.2 ± 1.3
	10	92.0 ± 1.5
3000	3	91.0 ± 2.5
	5	98.6 ± 2.8
	10	99.0 ± 2.3

Experimental parameters: sample volume 50 mL, Sm^3+^ 100 µg L^−1^, pH 2.5, aluminon 0.1 mmol L^−1^, Triton X-114 0.1% (v/v), KCl 0.1 mol L^−1^, at room temperature

### Selectivity

The impact of common concomitant ions on the extraction of Sm^3+^ (100 µg L^−1^) was investigated. The tolerable limits of interfering ions are summarized in Table 3. The tolerated level of each ion is the concentration that resulted in a recovery alteration of ± 5%. It has been shown that, under optimal conditions, the existence of other cations and anions at a certain ratio has no impact on AACPE of Sm^3+^. Therefore, the procedure is suitable for preconcentration of Sm^3+^ and its determination in real samples under the chosen conditions.

**Table 3:** Tolerance limits of concomitant species in the determination of Sm^3+^ (100 µg L^−1^)

Interfering ion	Interfering ion/analyte fold ratio (w/w)	Recovery (%)
Na^+^, K^+^, Mg^2+^, Ca^2+^, Cl-, NO_2_^−^, NO_3_^−^, SO_4_^2−^, HCO_3_^−^, CH_3_COO^−^, C_2_O_4_^2−^	< 2000	98.8 ± 1.5
Ba^2+^, Fe^2+^, Cu^2+^, Zn^2+^	1000	97.6 ± 2.2
Cd^2+^, Hg^2+^, Pb^2+^, Ni^2+^	500	97.9 ± 1.5
Ag^+^, Al^3+^, Fe^3+^, PO_4_^3−^	200	96.5 ± 3.1
Th^4+^, U^6+^, La^3+^, Ho^3+^, Er^3+^, Gd^3+^, Nd^3+^, Ce^4+^	50	95.0 ± 2.5

### Analytical figures of merits

Linearity, accuracy, limit of detection (LOD), limit of quantification (LOQ), and enrichment factor were used to evaluate the analytical performance of the optimized procedure. The calibration curve exhibited linearity across the concentration range of 0.2–200.0 µg L^−1^ for a sample volume of 50.0 mL. For 10 replicates measurements of 1.0 and 5.0 µg L^−1^ of Sm^3+^, the relative standard deviations (RSD) were 1.8 and 2.4%, respectively. The LOD and LOQ were 0.06 and 0.20 µg L^−1^, defined as the concentration of Sm^3+^ equivalent to three times and ten times the standard deviation of the blank divided by slope of the calibration graph, respectively. The enrichment factor was 102.0 when estimated as the ratio of the slopes of the calibration graphs with and without CPE. Table 4 compares the analytical characteristics of the presented study to those of other preconcentration procedures coupled with ICP-OES analysis. When compared to most existing methods, the suggested AACPE procedure performs better in terms of LOD and linearity. An additional advantage of the current procedure is rapidness. The heating step that is required for traditional CPE is useless in AACPE. It neither need stirring nor toxic solvents that are usually required for SPE.

**Table 4:** Comparison with other extraction procedure for determination of Sm^3+^ by ICP-OES

Preconcentration procedure	LOD (µg L^−1^)	Linearity (µg L^−1^)	EF	RSD (%)	Samples	Ref
Solid phase extraction onto multi-walled carbon nanotubes coated cellulose acetate membrane	0.20	0.5–100.0	201	2.6	Water and phosphoric acid	[5]
On-line surfactant-based solid phase extraction	0.50	2.0–100.0	92	2.9	Water and phosphoric acid	[35]
Mixed cloud point extraction using Alizarin Red S as complexing agent	0.09	0.3–100.0	97	3.2	Water and rock	[19]
Solid phase extraction using 1, 10- phenanthroline-2, 9-dicarboxilic acid modified Fe_3_O_4_/graphene oxide nanosheets	1.4	6.2–784.5	125	5.9	Water	[36]
Solid phase extraction using bentonite modified with N-(2-hydroxyethyl)ethylenediamine	0.6	Not provided	75	3.0	Wastewater	[37]
Ionic liquid-based dispersive liquid–liquid microextraction	1.3	Not provided	84	2.0	Uranium dioxide powders	[38]
Cloud point extraction using aluminon as complexing agent	0.06	0.2–200.0	102	1.8—2.4	Water and rock	The present study

### Analytical application

The proposed procedure for determination of Sm^3+^ was applied to a certified reference material (SRM 2780a) of Hard Rock Mine Waste to test its accuracy. The t value was calculated based on the following relation:

2 $$ t = \frac{X - X_o }{{s/\sqrt n }} $$

where *X* and *X*_*o*_ are the average measured and certified values for Sm^3+^, respectively, s is the standard deviation and n is the number of measurements. The results show the agreement between the measured value (4.6 ± 0.2 µg g^−1^) and certified one (4.7 µg g^−1^). Moreover, the *t* value (1.0) is smaller than the critical *t* value at 95 percent confidence (3.182) for degree of freedom of 3, indicating the accuracy of the procedure for the determination of Sm^3+^. The proposed AACPE procedure was used for trace analysis of Sm^3+^ by ICP-OES in wastewater and rock samples. Table 5 presented the analytical findings as well as the spiking sample analysis. The recovery (R) from spiked sample was determined using the following formula:

3 $$ R(\% ) = \frac{{{\text{Concentration}}_{{\text{spiked}}\,{\text{sample}}} - {\text{Concentration}}_{{\text{unspiked}}\,{\text{sample}}} }}{{{\text{Concentration}}_{{\text{added}}} }} $$

**Table 5:** Determination of Sm^3+^ in real samples by the proposed AACPE procedure (*n* = 5)

Sample	Added (µg L^−1^)	Found (µg L^−1^)	Recovery (%)	RSD (%)
Wastewater	0	Not detected	–	–
	2	1.96 ± 0.05	98.0	2.6
	5	4.94 ± 0.11	98.8	2.2
Rock	0	2.95 ± 0.08	–	2.7
	2	4.92 ± 0.13	98.5	2.6
	5	7.90 ± 0.23	99.0	2.9

As can be shown, the proposed approach quantitatively recovered the added Sm^3+^ from wastewater and rock samples (*R* = 98.0–99.0%). Furthermore, the RSD was less than 3.0%, showing high precision. These findings support the method's suitability for determining Sm^3+^ in wastewater and rock samples.

## Conclusion

A simple and innovative extraction technique, AACPE, was presented and combined, for the first time, with ICP-OES for preconcentration of Sm^3+^. The procedure is quick, precise, efficient, and sensitive. When compared to other preconcentration procedures such as SPE and traditional CPE, the extraction time is minimal. The procedure exhibits good analytical features for Sm^3+^ including low LOD, wide dynamic analytical range and acceptable preconcentration factor. Finally, the proposed approach was employed to determine Sm^3+^ in wastewater and rock samples at trace levels. We expect that the procedure will be used to extract other metal ions in different samples.

## Data Availability

All data generated during this study are included in the article.
